# Exploring polycystic disease solutions with ChatGPT: the role of AI in patient support and empowerment

**DOI:** 10.5339/qmj.2023.35

**Published:** 2024-01-10

**Authors:** Jackson Keefer Jerry

**Affiliations:** ^1^Internal Medicine, PSG Institute of Medical Sciences and Research, India Email: jkjerry 2023@gmail.com

**Keywords:** polycystic ovarian disease, artificial intelligence, ChatGPT, gynecology

OpenAI, headquartered in San Francisco, is an artificial intelligence (AI) research and development company focused on creating safe AI software for the betterment of humanity.^1^ Among their creations is the Generative Pre-trained Transformer (ChatGPT), a chatbot introduced by OpenAI in November 2022.^1^ Utilizing language processing and machine learning, ChatGPT allows users to engage in conversational interactions with a virtual assistant.^2^

ChatGPT has garnered worldwide attention by producing lifelike and cleverly crafted text that can often be mistaken for content written by humans. In a study, 32% of the generated abstracts were incorrectly identified by human reviewers as genuine, as ChatGPT’s output skillfully evaded plagiarism checks. These technical advances raise ethical concerns in scientific research, where accurate and evidence-based information is essential. The lack of automated tools to verify the originality and accuracy of ChatGPT’s abstracts compounds these issues.^3^

Given the significant attention ChatGPT has been receiving, the general public will soon turn to it to explore personal gynecological concerns. A recent study revealed that ChatGPT provided high-quality and empathetic responses to patient queries raised in an online forum.^5^

Polycystic ovary syndrome (PCOS) is a heterogeneous, chronic endocrine disorder commonly found in women of reproductive age. It is characterized by various symptoms such as menstrual dysfunction, infertility, hirsutism, acne, and obesity. To identify PCOS, three sets of criteria have been established: the National Institutes of Health criteria (1992), Rotterdam criteria (2003), and Androgen Excess Society criteria (2006). All three subsets require the presence of chronic oligo/anovulation, clinical and/or biochemical hyperandrogenism, and polycystic ovarian morphology on transvaginal ultrasound or combinations of these conditions.^5^ Before confirming the diagnosis of PCOS, other disorders that can cause anovulation and/or androgen excess must be ruled out. Some researchers and clinicians rely on menstrual cycles to determine ovulatory status, defining oligo or anovulation as menstrual cycles lasting less than 21 or more than 35 days. Polycystic ovarian morphology is considered significant when there are ≥12 follicles with a 2-9 mm diameter or an ovarian volume of >10 ml.^5^

Individuals at risk of PCOD are increasingly relying on AI, like ChatGPT and other feasible options, to acquire information about PCOD and seek potential remedies for the condition. By doing so, they can reduce the occurrence and indulge in a lifestyle to avoid it.

The attached table involves a series of questions that a person without access to analog medical knowledge can raise.

As per our observation, ChatGPT generates detailed write-ups with essentially correct information in easy-to-understand language, such as “hormonal imbalance can cause excess facial and body hair growth(Hirsutism).” It is interesting to observe that ChatGPT conveys realistic information such as “PCOD is a chronic condition that currently does not have a cure. However, it can be effectively managed” and directs users to “consult a healthcare professional for proper diagnosis.” It also generates information such as over-the-counter medications like ‘Salicylic Acid’ used to treat PCOD-induced acne and avoiding the use of ‘Buserelin’ in pregnancy.

While ChatGPT offers empathetic responses to PCOD patients’ concerns, the lack of verification for these responses challenges their authenticity, as they do not include any cited sources or references to validate the information.

While language learning models (LLMs) such as ChatGPT hold promise for clinical information retrieval in the future, they have a limitation in that their responses are non-deterministic. Minor changes in input can lead to vastly different responses. Nevertheless, ChatGPT can aid gynecologists in promptly offering empathetic and high-quality information to address patient concerns through online platforms and consultations. Gynecologists can also monitor and regulate this information by cross-referencing it with reliable sources like medical journals, thereby ensuring the dissemination of accurate and verified information.

Additional research should focus on quantifying the reliability and accuracy of patient responses generated by ChatGPT. Collaboration among gynecologists interested in AI could potentially result in developing new AI software based on LLMS. This software would be able to produce verified responses of superior quality and reliability in addressing patient queries.

## Conflicting Interest

No conflict of interest to declared.

## Acknowledgments

The authors acknowledge the guidance of The Good Research Project towards the successful completion of this research and manuscript writing.

## Figures and Tables

**Table:** 

No.	Question	ChatGPT Response
1.	What is PCOD?	PCOD stands for Polycystic Ovary Syndrome, which is a common hormonal disorder affecting women of reproductive age. It is also known as Polycystic Ovarian Syndrome (PCOS). PCOD is characterized by a combination of signs and symptoms related to hormonal imbalances and ovarian dysfunction.
2.	What are the clinical features of PCOD?	Polycystic Ovary Syndrome (PCOS), also known as Polycystic Ovary Disease (PCOD), is a common hormonal disorder that affects women of reproductive age. The clinical features of PCOD can vary from woman to woman, and the severity of symptoms can also differ. To be diagnosed with PCOD, a woman typically needs to have at least two of the following three features: • Menstrual Irregularities: Women with PCOD often experience irregular menstrual cycles. This can manifest as oligomenorrhea (infrequent or irregular periods) or amenorrhea (absence of menstruation). • Hyperandrogenism: Elevated levels of androgens (male hormones) are a hallmark of PCOD. This can lead to various signs of androgen excess, including: • Hirsutism: Excessive hair growth on the face, chin, chest, abdomen, or other body parts, similar to male pattern hair growth. • Acne: Severe acne, often along the jawline, chin, and lower cheeks. • Male-pattern baldness: Thinning hair or hair loss similar to male-pattern baldness. • Polycystic Ovaries: On ultrasound examination, the ovaries may appear enlarged and contain multiple small follicles (cysts). However, it’s essential to note that the presence of polycystic ovaries alone is not sufficient for a PCOD diagnosis, as many women may have polycystic ovaries without exhibiting the other symptoms. Other clinical features and associated symptoms of PCOD may include: • Weight gain or difficulty losing weight: PCOD is associated with insulin resistance, leading to weight gain and difficulty maintaining a healthy weight. • Fatigue and low energy levels. • Mood swings and emotional disturbances. • Skin tags (small, flesh-colored growths) in the armpits or neck. • Darkening of the skin in certain areas (acanthosis nigricans), such as the neck, underarms, and groin. • Infertility or difficulties in getting pregnant due to irregular ovulation. It’s crucial to remember that PCOD can present differently in different individuals, and not all women with PCOD will exhibit all the clinical features mentioned above. Additionally, these symptoms can overlap with other medical conditions, so it’s essential to consult a healthcare professional for an accurate diagnosis and appropriate management. A proper evaluation by a healthcare provider may involve a medical history review, physical examination, blood tests to check hormone levels, and ultrasound imaging of the ovaries.
3.	What causes PCOD?	The exact cause of Polycystic Ovary Syndrome (PCOS) or Polycystic Ovary Disease (PCOD) is not entirely understood. It is a complex and multifactorial condition influenced by both genetic and environmental factors. Several underlying mechanisms have been proposed, and these factors can vary among individuals. The primary factors believed to contribute to PCOD include: • Hormonal Imbalance: PCOD is characterized by an imbalance in reproductive hormones, particularly elevated levels of androgens (male hormones) such as testosterone. This hormonal imbalance disrupts the normal development and release of eggs from the ovaries, leading to the formation of multiple small follicles (cysts). • Insulin Resistance: Insulin is a hormone that helps regulate blood sugar levels. Insulin resistance occurs when cells in the body become less responsive to the effects of insulin. This condition is closely linked to PCOD and contributes to the increased production of androgens by the ovaries. Insulin resistance can also lead to weight gain and difficulty in losing weight, as the body may produce more insulin to compensate for the resistance, and insulin itself can promote androgen production. • Genetics: PCOD tends to run in families, suggesting a genetic component. If you have a family history of PCOD, you may have an increased risk of developing the condition. • Inflammation: Chronic low-grade inflammation in the body may play a role in PCOD, as it can affect insulin sensitivity and hormone production. • Lifestyle Factors: Poor diet, lack of physical activity, and obesity can contribute to insulin resistance, which, in turn, exacerbates PCOD symptoms. • Environmental Factors: Exposure to certain environmental factors, such as endocrine-disrupting chemicals, may also influence hormonal balance and contribute to PCOD development. It’s important to understand that PCOD is a complex and heterogeneous condition, and the interplay of these factors can vary from person to person. While we know the contributing factors, the precise triggering mechanism for PCOD remains a subject of ongoing research. Diagnosing PCOD involves a comprehensive evaluation by a healthcare professional, including a medical history review, physical examination, blood tests to assess hormone levels, and an ultrasound to visualize the ovaries and assess their appearance. Treatment options for PCOD focus on managing symptoms, improving hormonal balance, and addressing associated health concerns like insulin resistance and obesity. Lifestyle changes, such as adopting a healthy diet and regular exercise, are often recommended in managing PCOD symptoms. In some cases, medications may be prescribed to regulate hormones and improve fertility.
4.	What is the medical management of PCOD?	The medical management of PCOD (Polycystic Ovary Syndrome) involves a combination of lifestyle changes and medications. The specific treatment plan may vary based on individual symptoms and health needs. It is essential to consult a healthcare professional, such as a gynecologist or endocrinologist, for proper diagnosis and personalized treatment. Here are some common medical interventions for PCOD: • Birth Control Pills: Oral contraceptives are often prescribed to regulate menstrual cycles and reduce androgen levels. They can help improve hirsutism (excess hair growth) and acne. • Anti-Androgen Medications: Certain medications, such as spironolactone, can block the effects of androgens and help reduce symptoms like hirsutism and acne. • Metformin: This medication is commonly used to treat type 2 diabetes, but it can also be beneficial for women with PCOD, especially those with insulin resistance. Metformin helps improve insulin sensitivity and may regulate menstrual cycles. • Ovulation Induction: For women trying to conceive, medications like clomiphene or letrozole may be prescribed to stimulate ovulation. • Gonadotropins: In some cases, injectable hormones called gonadotropins may be used to induce ovulation when other medications are not effective. • Lifestyle Modifications: As part of medical management, healthcare providers often recommend lifestyle changes, including a balanced diet, regular exercise, and weight management, to improve insulin sensitivity and hormonal balance. • Surgical Intervention: In some cases, ovarian drilling may be considered to induce ovulation in women who have not responded to other treatments. However, this is usually a last resort and not a common approach. It’s important to note that PCOD is a chronic condition, and its management may require ongoing monitoring and adjustments to the treatment plan based on the individual’s response. The primary goals of medical management are to regulate menstrual cycles, reduce androgen levels, improve fertility (if desired), and manage associated symptoms and health risks. Additionally, women with PCOD may have an increased risk of other health conditions, such as type 2 diabetes, high blood pressure, and heart disease. Regular check-ups and screenings are essential to monitor and manage any potential complications.
5.	How to prevent PCOD?	• Maintain a Healthy Weight: Being overweight or obese is a significant risk factor for PCOD. Adopting a balanced and nutritious diet and engaging in regular physical activity can help in maintaining a healthy weight or achieving weight loss if necessary. • Birth Control Pills: For some women at higher risk of PCOD, birth control pills can be prescribed to control symptoms. • Exercise Regularly: Regular physical activity can improve insulin sensitivity and hormonal balance, which may reduce the risk of developing PCOD or help manage existing symptoms. • Avoid Crash Diets: Extreme or crash diets can disrupt hormonal balance and may lead to irregular menstrual cycles. Focus on sustainable and balanced eating patterns. • Manage Stress: Chronic stress can impact hormone levels and contribute to the development of PCOD. Practice stress-reduction techniques such as meditation, yoga, or engaging in hobbies that bring joy and relaxation. • Limit Processed Foods and Sugar: Reducing the intake of processed foods, sugary snacks, and beverages can help regulate insulin levels and support hormonal balance. • Be Mindful of Carbohydrates: Choose complex carbohydrates (whole grains, fruits, and vegetables) over simple carbohydrates (sugary foods and drinks) to help manage insulin levels. • Avoid Smoking and Excessive Alcohol Consumption: Both smoking and heavy alcohol consumption can have adverse effects on hormonal balance and overall health. • Regular Health Check-ups: Regular medical check-ups can help identify and manage any underlying health conditions that may contribute to PCOD risk. • PCOD due to a family history or other risk factors, taking oral contraceptive pills may be recommended as a preventive measure to regulate hormone levels and menstrual cycles. • Early Detection and Treatment: If you have irregular menstrual cycles or suspect symptoms of PCOD, consult a healthcare professional for early diagnosis and appropriate management. Early intervention can help prevent long-term complications and improve overall health.
6.	Can I use metformin to treat PCOD?	Metformin is a medication commonly used to treat type 2 diabetes, but it is also sometimes prescribed off-label for certain cases of PCOD (Polycystic Ovary Syndrome). Metformin may be used in women with PCOD, especially if they have insulin resistance or impaired glucose tolerance, as it can help improve insulin sensitivity and hormonal balance.
7.	Can buserelin be used to treat PCOD?	Buserelin is not typically used as a primary treatment for PCOD (Polycystic Ovary Syndrome). Buserelin is a medication that belongs to a class of drugs known as Gonadotropin-Releasing Hormone (GnRH) agonists. It is primarily used in the management of certain reproductive and gynecological conditions, such as endometriosis, and assisted reproductive technologies (ART) like in vitro fertilization (IVF). While buserelin and other GnRH agonists can affect hormone levels, they are not typically considered the first-line treatment for PCOD. In some specific cases, GnRH agonists may be used to induce ovulation or temporarily suppress ovarian function, but this is not the standard approach for managing PCOD.
8.	Can I use Buserelin for PCOD, if I am pregnant?	Buserelin is not recommended for use during pregnancy. During pregnancy, hormonal treatments like Buserelin are not appropriate because they can interfere with the natural hormonal changes necessary for a healthy pregnancy. Additionally, Buserelin is not intended for the treatment of PCOD (Polycystic Ovary Syndrome) as a primary therapy. Can PCOD be cured? As of my knowledge cutoff in September 2021, PCOD (Polycystic Ovary Syndrome) is a chronic condition that currently does not have a cure. However, it can be effectively managed with a combination of lifestyle changes and medical treatments, allowing women with PCOD to lead healthy and fulfilling lives.
9.	Can Steroid creams be used to treat acne in PCOD?	Steroid creams are generally not recommended for the treatment of acne in women with PCOD (Polycystic Ovary Syndrome). Acne in PCOD is primarily caused by the increased production of androgens (male hormones) in the body, leading to excessive sebum production and clogged pores. Steroid creams, also known as topical corticosteroids, are anti-inflammatory medications primarily used to treat various skin conditions, such as eczema, psoriasis, and allergic reactions. While they can be effective in reducing inflammation, they are not designed to address the underlying hormonal imbalances that cause acne in PCOD. Moreover, the prolonged use of topical corticosteroids on the face can lead to potential side effects, including skin thinning, increased susceptibility to infections, and the development of steroid-induced acne (acneiform eruptions). Instead of steroid creams, the treatment of acne in PCOD usually involves the following approaches: • Topical Acne Treatments: Over-the-counter or prescription topical treatments containing ingredients like benzoyl peroxide, salicylic acid, or retinoids can be used to unclog pores, reduce inflammation, and control acne. • Oral Medications: Oral antibiotics or oral contraceptives (birth control pills) may be prescribed to help control acne and reduce androgen levels. • Anti-Androgen Medications: In some cases, anti-androgen medications like spironolactone may be prescribed to block the effects of androgens on the skin, reducing acne and excess hair growth. • Skin Care Routine: A gentle and consistent skincare routine can help keep the skin clean and reduce acne breakouts. • Lifestyle Modifications: Maintaining a healthy diet, regular exercise, and managing stress can also contribute to managing acne in PCOD.
10.	Can PCOD be cured?	As of my knowledge cutoff in September 2021, PCOD (Polycystic Ovary Syndrome) is a chronic condition that currently does not have a cure. However, it can be effectively managed with a combination of lifestyle changes and medical treatments, allowing women with PCOD to lead healthy and fulfilling lives.

## References

[1] OpenAI [Internet] About OpenAI (c2015-2023). https://openai.com/about/.

[2] King MR (2023). chatGPT. A Conversation on Artificial Intelligence, Chatbots, and Plagiarism in Higher Education. Cell Mol Bioeng.

[3] Else H (2023). Abstracts written by ChatGPT fool scientists. Nature.

[4] Ayers JW, Poliak A, Dredze M, Leas EC, Zhu Z, Kelley JB (2023). Comparing Physician and Artificial Intelligence Chatbot Responses to Patient Questions Posted to a Public Social Media Forum. JAMA Intern Med.

[5] Rao M, Broughton KS, LeMieux MJ (2020). Cross-sectional Study on the Knowledge and Prevalence of PCOS at a Multiethnic University. Progress in Preventive Medicine.

